# Supplementary material for: An evidence based efficacy and safety assessment of the ethnobiologicals against poisonous and non-poisonous bites used by the tribals of three westernmost districts of West Bengal, India: Anti-phospholipase A2 and genotoxic effects
Source: PLoS One. 2020 Nov 30;15(11):e0242944. doi: 10.1371/journal.pone.0242944 (PMC7703885; doi:10.1371/journal.pone.0242944)
Supplement: S1 Fig — (DOCX) [file pone.0242944.s001.docx]

**Fig S1. Interview data sheet**

| **INTERVIEW DATA SHEET** |
| --- |
| **Locality (village etc.):**  **Date:** |
| **Details of traditional medicine men (TMM):** |
| Name: |
| Address: |
| Age: |
| Gender: |
| Ethnicity: |
| Years of experience: |
| **Field study:** |
| Location, time, date and season of sample collection: |
| **Details of botanicals:**  Local name(s):  Binomials:  Family:  Habit:  Habitat:  Identifying characters:  **Details of zoologicals:**  Local name(s):  Binomials:  Family:  Identifying characters:  **Traditional healing:**  Medical condition treated:  Used as (monoherbal/polyherbal):  Use of additives/taste enhancers (if any):  Plant/animal part used:  Quantity:  Method of preparation:  Mode of administration:  Dose:  Side effects (if any):  Restrictions during treatment (if any): |
