# Supplementary figures and images for: An evidence based efficacy and safety assessment of the ethnobiologicals against poisonous and non-poisonous bites used by the tribals of three westernmost districts of West Bengal, India: Anti-phospholipase A2 and genotoxic effects
Source: PLoS One. 2020 Nov 30;15(11):e0242944. doi: 10.1371/journal.pone.0242944 (PMC7703885; doi:10.1371/journal.pone.0242944)

**Fig S2. Specimen copy of an interview data sheet**


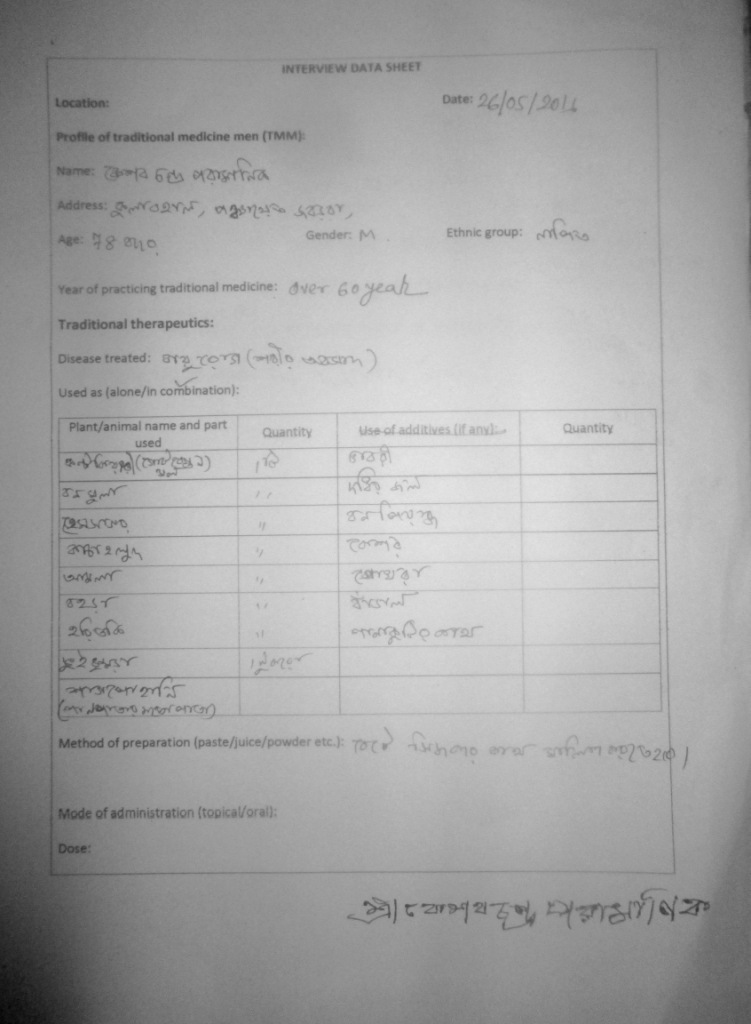

Supplement: S2 Fig — (DOCX) [file pone.0242944.s002.docx]

**Fig S3. A consent letter in Bengali language provided by one of the informants**


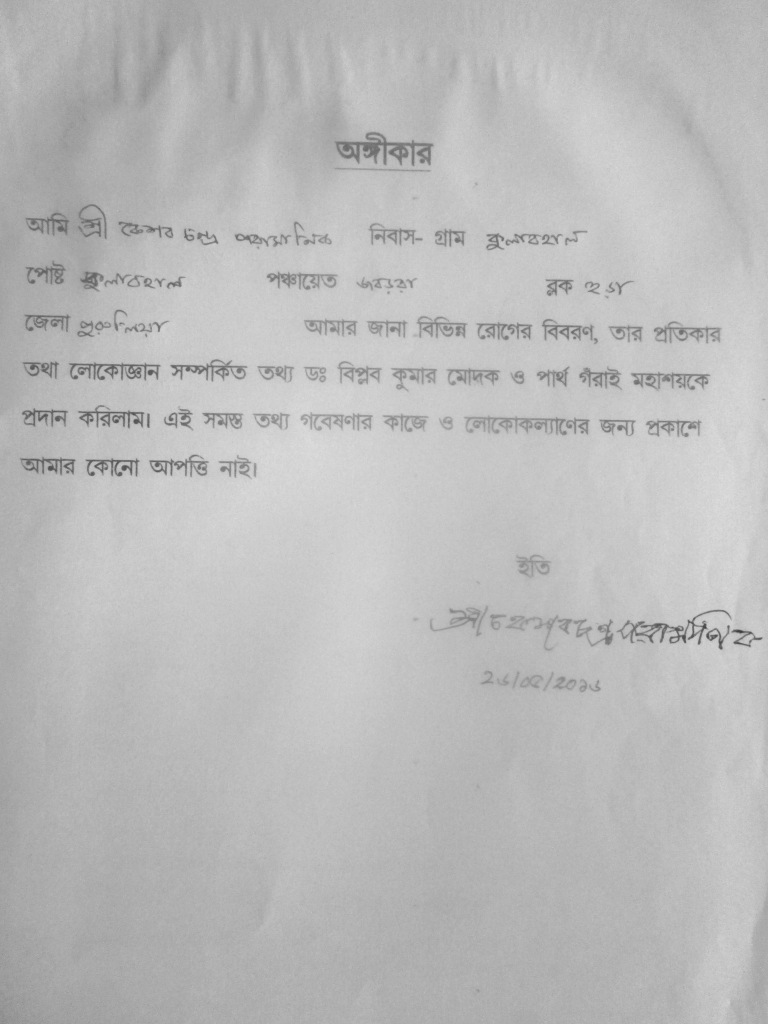

Supplement: S3 Fig — (DOCX) [file pone.0242944.s003.docx]
