# Supplementary material for: An evidence based efficacy and safety assessment of the ethnobiologicals against poisonous and non-poisonous bites used by the tribals of three westernmost districts of West Bengal, India: Anti-phospholipase A2 and genotoxic effects
Source: PLoS One. 2020 Nov 30;15(11):e0242944. doi: 10.1371/journal.pone.0242944 (PMC7703885; doi:10.1371/journal.pone.0242944)
Supplement: S1 Graphical abstract — (PPTX) [file pone.0242944.s004.pptx]

## Slide 1
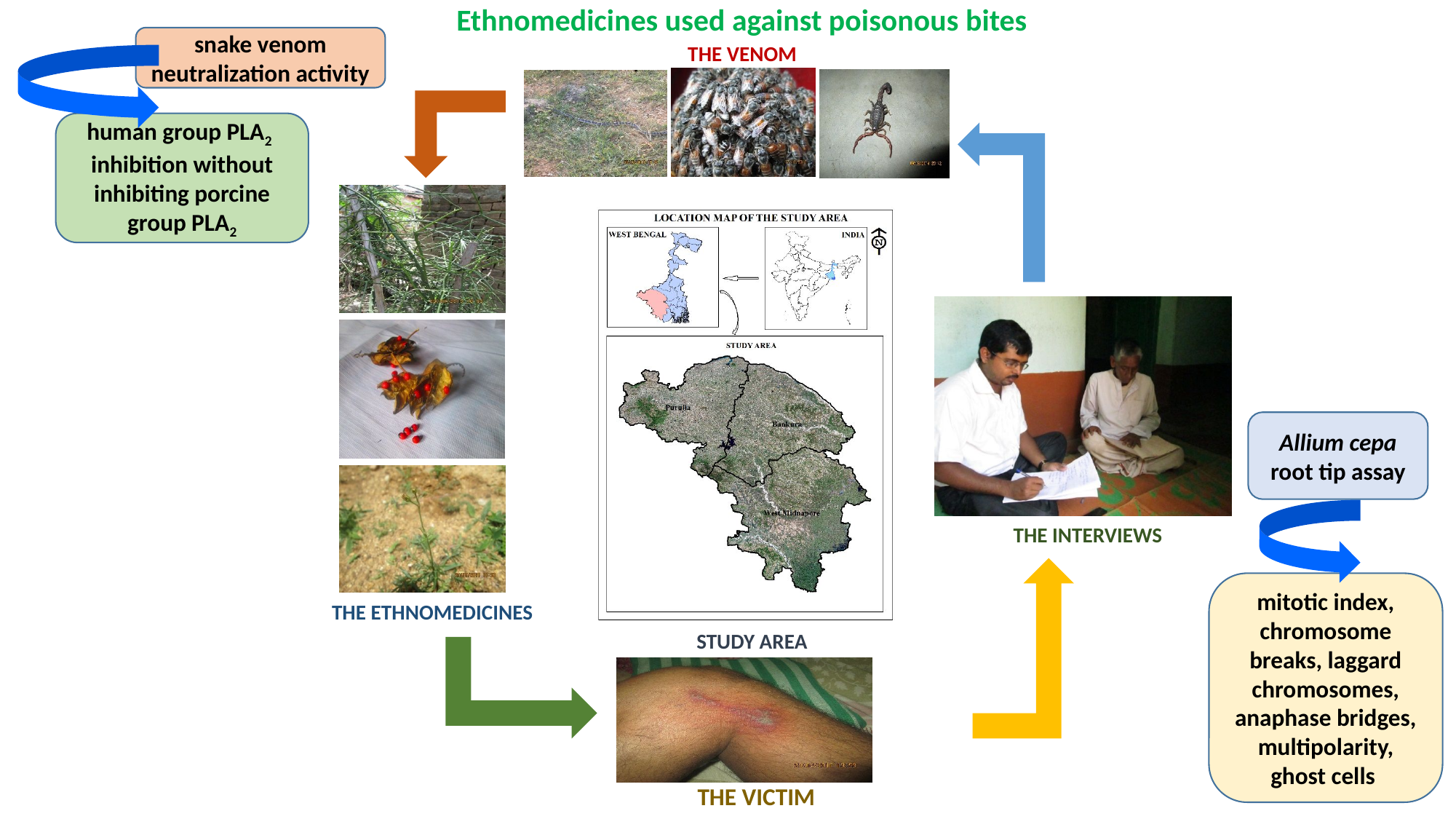

Ethnomedicines used against poisonous bites
snake venom neutralization activity
THE VENOM
human group PLA2 inhibition without inhibiting porcine group PLA2
Allium cepa root tip assay
THE INTERVIEWS
mitotic index, chromosome breaks, laggard chromosomes, anaphase bridges, multipolarity, ghost cells
THE ETHNOMEDICINES
STUDY AREA
THE VICTIM
