# Supplementary material for: An evidence based efficacy and safety assessment of the ethnobiologicals against poisonous and non-poisonous bites used by the tribals of three westernmost districts of West Bengal, India: Anti-phospholipase A2 and genotoxic effects
Source: PLoS One. 2020 Nov 30;15(11):e0242944. doi: 10.1371/journal.pone.0242944 (PMC7703885; doi:10.1371/journal.pone.0242944)
Supplement: Attachment — Submitted filename: Response to reviewers.docx [file pone.0242944.s005.docx]

**Authors’ response to the comments:**

All changes are in track change mode in the revised manuscript. In the track change mode, we have also changed the tables from portrait to landscape (as directed by the reviewers).

| **Queries** | **Answers** |
| --- | --- |
| To ensure that you obtained ethics approval before your study began, please upload the ethics approval document issued by West Bengal Biodiversity Board approval (1.1.2008-22.5.2009) [Memo No. 5k(bio)-2/2007].  Please upload the completed Content Permission Form or other proof of granted permissions as an "Other" file with your submission. | Thank you. The desired documents are attached. The document of project [Memo No. 5k(bio)-2/2007] carried out is approved by the Principal, Acchuram Memorial College, under Sidho Kanho Birsha University, the then institute of the first author. The same document also confirms the other projects carried out in this regard. The second document from West Bengal Biodiversity Board, a Govt. Body also confirms the same project [Memo No. 5k(bio)-2/2007] on “Preparation of people’s biodiversity register of Jhalda-Darda Gram Panchayat” carried out by the first author. This was issued by Sr. Research Officer, West Bengal Biodiversity Board. Authors having all these documents in original and can exhibit if needed. Permission Form or other proof of granted permissions as an "Other" file being submitted. |
| We note that [Figure(s) 1] in your submission contain [map/satellite] images which may be copyrighted. We require you to either (1) present written permission from the copyright holder to publish these figures specifically under the CC BY 4.0 license, or (2) remove the figures from your submission: | Map was created using the editor tools of the ArcGIS 10.3.1 software. It is mentioned now in the caption of Fig. 1. |
| **Reviewer #1:** |  |
| The article “An evidenced based efficacy and safety assessment of the antivenom ethnobiologicals used by the tribals of three westernmost districts of West Bengal, India: Anti-phospholipase A2 and genotoxic effects.” presents a survey conducted with traditional healers from a specific region in India, aiming to catalogue traditional formulations used as treatments against animal bites and stings and assess their efficacy and safety using experimental assays with the reported ethnobotanicals. It is an interesting work that addresses an important matter because not only the traditional knowledge can drive further investigations on the pharmacological potential of the natural products, leading to new drugs, but it is also important to characterize possible toxic effects of such formulations in order to protect the population making use of them. Considering the neglected issue of envenoming and the scarcity of available treatments, and the fact that the populations living in poor and isolated districts are mostly affected by these problems, the presented article has merit.  I would recommend this work for publication in PLOSOne, but it must be submitted to a major revision, in order to attend the journal’s quality standards. First of all, the manuscript is to long. Information should be presented in a more concise and direct language, highlighting the main findings. For this, English language, although intelligible, must also be revised. | Thank you for your kind comments. Now after the revision, we have tried to use concise and direct language which has also been revised extensively. |
| Other issues are listed below:  Title: the work title mentions “antivenom ethnobiologicals”, but dog/cat/hyena bites, which do not contain venom, are listed as ailments for the traditional formulations. Whether the title should be modified or these particular conditions must be taken out from the results. | Thank you for your kind comments. It is corrected as suggested by you. |
| The interviewees are mentioned as “traditional healers” (TH) and “traditional medicine man” (TMM) along the text. This can cause confusion, so only one of the terms should be chosen. | Thank you for your kind comments. It is corrected as suggested by you. |
| Material and Methods: in the “Study area and aboriginals” session, the authors make narrative description that, although interesting, should be better placed in the discussion session. The Material and Methods should deliminate the study subject in more direct form. | Thank you for your kind comments. It is corrected as suggested by you. |
| The period of time when the survey was conducted differs between the ‘Abstract’ (2008-2009 and 2010-2017) and ‘Materials and Methods’. Which one is correct? | Thank you for your kind comments. It is corrected as suggested by you. |
| Table 1 would be better placed in the ‘Results session’. | Thank you for your kind comments. It is corrected as suggested by you. |
| In the Results session, it would be interesting to have the demographic profile of the interviewees (sex/age/profession/district). | Thank you for your kind comments. It is corrected as suggested by you. |
| The second session in ‘Results’ is named ‘Preparations, application and dose-dependence of TFs’, but there is no mention of the dose-dependence in the results. | Thank you for your kind comments. It (dose dependence) is deleted as suggested by you. |
| Table 2 should me made horizontally, to increase readability and should be placed in the ‘Quantitative ethnobiology’ session. | Thank you for your kind comments. It is corrected as suggested by you. |
| Also, in this table, what does “voucher number’ stands for? And the numbers that appear after the description of ‘parts used’? This information should be removed or explained in the table’s legend. | Thank you for your kind comments. It is corrected as suggested by you. Voucher no. and the numbers that appear after the description of ‘parts used’ are explained in the footnote. |
| Information on abbreviations is also lacking in table 5. | Thank you for your kind comments. It is added as suggested by you. |
| As a suggestion, data presented in table 6 would be more clearly visualized as a Graph. | Thank you for your kind comments. It is corrected as suggested by you.Table 6 is replaced by |
| Table 7 would also be better read horizontally. | Thank you for your kind comments. It is corrected as suggested by you. |
| The Discussion session, unlike the other session, is rather succinct. I believe the other parts of the manuscript could be more concise and Discussion should be further explored, comparing the found information with other similar articles, performed in other regions. | Thank you for your kind comments. It is corrected as suggested by you.  Discussion part is further elaborated comparing the found information with other similar articles, performed in other regions. |
| In line 419, authors state that ‘Most of the snake bites are either dry bites or from non-Poisonous snakes. Still death occurs. There are many instances where patients die only because of fear and psychological shock.’ This seemed odd to me. Are there any references to support this statement? | Thank you for your kind comments. References are added to support this statement. |
| In line 494 of the ‘Conclusion’, authors claim that ‘Various conservation strategies have also been described as adopted by the indigenous people in order to use plant resources sustainably.’. I did not find the description of these strategies throughout the text. | Thank you for your kind comments. This part is deleted and rewritten. |
| Reviewer #2: |  |
| A broad review regarding the English language, punctuation and spacing should be made. | Thank you for your kind comments. We have corrected the language with the best of our ability. |
| Several references are mentioned incorrectly during the text, such as seen on lines 102, 105 and 109, | Thank you for your kind comments. It is corrected as suggested by you. |
| During introduction, authors stress that PLA2 enzymes are responsible for the inflammatory effects of snake bites, and that plant extracts detain some sort of PLA2 neutralizing capacity. However, activity of PLA2 of both human and porcine origin are also evaluated. These subgroups must be more clearly determined in methodology and results and discussion.  Authors should classify the different Authors also claim to evaluate genotoxicity and cytotoxicity, and that should be clear as an aim of the present study as well. | Thank you for your kind comments. It is corrected as suggested by you. We have added portions in the methodology and result sections where the subgroups are more clearly determined. We have also added a number of references in the discussion to classify the different Authors also claim to evaluate genotoxicity and cytotoxicity. |
| The aim of the study is not clearly outlined by the authors. | Thank you for your kind comments. The aim of the study is now clearly added at the end of introduction section. |
| On Table 1, TF4 is explained as masticated by hand, a term with dubious meaning. | Thank you for your kind comments. It was corrected and replaced. |
| Table 2 is impossible to read and contains some of the main results from the experiment that should be clearly available to the reader. | Thank you for your kind comments. The table was reframed to be clearly available to the reader. |
| Table 7 also needs formatting. | Thank you for your kind comments. As suggested by the first reviewer we have replaced the data of Table 7 with Figure 7 presenting the same dataset. |
| Discussion is very superficial, mainly replicating results obtained. It should be rewritten, stressing what each plant extract detains from a biochemical or pharmacological level that corroborates with a lesser or more prominent effect regarding PLA2 neutralization. | Thank you for your kind comments. The discussion part is elaborated explaining the plant extract from a biochemical or pharmacological level with a number of newly added references which corroborates with a lesser or more prominent effect regarding PLA2 neutralization. |
| Since no aim of the study was properly established, conclusion is also in need of revision. | Thank you for your kind comments. We have rewritten the aim of the study and hence the conclusion is also rewritten accordingly. |
