# Supplementary material for: An evidence based efficacy and safety assessment of the ethnobiologicals against poisonous and non-poisonous bites used by the tribals of three westernmost districts of West Bengal, India: Anti-phospholipase A2 and genotoxic effects
Source: PLoS One. 2020 Nov 30;15(11):e0242944. doi: 10.1371/journal.pone.0242944 (PMC7703885; doi:10.1371/journal.pone.0242944)
Supplement: Attachment — Submitted filename: Response to reviewers.docx [file pone.0242944.s006.docx]

**Authors’ response to the comments:**

**As needed Figures S1, S2 and S3 are included in this version of submission.**

All changes are in track change mode in the revised manuscript. In the track change mode.

| **Reviewer #1:** |  |
| --- | --- |
| **Queries** | **Answers** |
| Although the manuscript has been consistently improved from the first submission, there are still some points that should be further addressed prior to publication: | Thank you. |
| In Table 1, there are still unexplained numbers, placed after "Disease/ Disorder" and after some words like "ointment (1070000)". These numbers should be further explained in the legend or footnote.  In addition, the units abbreviations described in the TF Composition (eg: Seowra (Streblus asper) leaves: 4-5 in no.) should also be described in the table's footnotes. | The numbers used in parenthesis after the diseases/disorders, parts and methods of administration in table 1 and 2 are according to the recommendations given by Cook, 1995 as Economic botany data collection standard (EBDCS), plant parts, body parts and processes, disorders/effects, medicinal applications and non-vertebrate organisms (Master lists of states for Level 3 descriptors) (Economic Botany Data Standard; https://www.kew.org/tdwguses/rptMasterListMain.htm). 3In composition, in no. used after the numbers stands for in number i.e. the number of that plant part used.  This information is added to the footnote in both the tables.  In addition, the units abbreviations described in the TF Composition (eg: Seowra (Streblus asper) leaves: 4-5 in no.) are also described in the table's footnotes. |
| Likewise, for Table 2, numbers placed after "Parts used" (eg: root (12040000)), and "Ailment treated" are not explained neither in the table's legend nor in the footnote. | The numbers used in parenthesis after the diseases/disorders, parts and methods of administration in table 1 and 2 are according to the recommendations given by Cook, 1995 as Economic botany data collection standard (EBDCS), plant parts, body parts and processes, disorders/effects, medicinal applications and non-vertebrate organisms (Master lists of states for Level 3 descriptors) (Economic Botany Data Standard; https://www.kew.org/tdwguses/rptMasterListMain.htm). 3In composition, in no. used after the numbers stands for in number i.e. the number of that plant part used.  This information is added to the footnote in both the tables. |
| The session "Use of ethnobotanicals: toxicity, conservation status and Economic Botany Data Standard" is still a bit confusing, as different subjects are discussed in the same paragraph, without an evident connection between them. | Yes, that is why we removed the irrelevant Economic Botany Data Standard from this section, explaining as footnotes in table 1 and 2 as suggested by the reviewers. This section of the result is now renamed as “Use of ethnobotanicals: toxicity, aspects and conservation status” in accordance to the section in the discussion “Use of plants and animals as drugs: toxicity and conservation aspects” |
